# The Cerebellum

**Published:** 1909-07-17

**Authors:** Victor Horsley


### THE CEREBELLUM.

By Sir VICTOR HORSLEY, F.R.S., F.R.C.S.

(A Brief Abstract of the Cavendish Lecture delivered to the West London Medico-Chirurgical Society on June 25, 1909.)

The conception of the cerebellum as an organ governing and co-ordinating the muscular response to stimuli received through the sensory nerves was originated by Willis in the seventeenth century. Experiments upon animals have shown that, unless the cerebellum is intact, running and walking, and even standing, are impossible. The automatic function of standing involves extension, of which the newly born of most mammals are incapable. The new-born infant and the new-born kitten can neither of them stand because they have not the necessary power of extending their limbs. In the new-born kitten a section of the cerebellum shows that the molecular layer is still quite embryonic: the Purkinje's bodies are there, but the cells of the molecular layer over the whole of the cerebellum are in an embryonic state.

At the same time we do not stand up wholly through the existence of our cerebellum, nor does any other animal. We stand erect upon our hind legs, and we do so by means of the extensors of the knees, hips, and ankles chiefly. There are other important centres besides those of the cerebellum concerned in this. Galen recognised that the spinal cord of a lower animal is a mass of centres. This was first demonstrated experimentally by Munk, and Sherrington has shown the details of how this function is subserved. It is the afferent impulses brought to the cerebellum through the various spinal centres which enable the standing position to be maintained without effort. It is often said in text-books that the ataxia of the tabetic is due to sclerosis of the posterior columns of the cord. But it has been shown that in animals both the posterior columns can be cut across without any ataxia resulting: ataxia always results when the posterior nerve-roots are cut.

To pass from standing to walking, we still really walk with all four legs: the swing of the arms alternates with that of the legs, which Dr. Hughlings Jackson, the father not only of British neurology, but of all neurology, calls the alternation of corresponding opposites. This is, of course, because we are each of us two individuals joined together in the middle line.

If you stimulate the region of the thalamus to which the superior cerebellar peduncle is distributed, you get a movement of progression. Dr. Jackson published an article in *Brain* two years ago, illustrated with drawings by Dr. Mackenzie of an extreme and untreated case of post-basic meningitis. These drawings may some day possess a historic interest, because in the future it may not be possible to see such cases of extreme cerebro-spinal meningitis: the patients will be treated and cured before they reach such a condition. These drawings show, besides head retraction, the alternating extension and flexion of the arms and legs, as in the act of walking. If we consider running, which is nothing but exaggerated walking, the alternation of corresponding opposites is shown extremely well, as, for instance, in a photograph of the finish of a running-race.

On stimulation of the whole cerebellum the effect produced upon the eyes is one of conjugate deviation. Turning to the labyrinth and semi-circular canals, the special afferent apparatus of equilibration is found. It is not a static apparatus, but is constructed wholly upon the idea that movement is going on. The movement of the fluid in the canals produces a purely mechanical stimulation of the cells.

The "cerebellar" attitude of the head, so often of use in the diagnosis of cerebellar disease, should really be called the "vestibular" attitude, for it is. I suggest, really due to lesions of the labyrinth. This is not in any way to detract from its value as a sign in cerebellar cases. As a rule the head in this condition inclines to the affected side: unfortunately there are exceptions to this, and some of them have lately been creeping into text-books as the usual rule instead of the exceptions to it. If you remove the labyrinth in mammals, the result is always inclination of the head to the side of the lesion.

I wish to conclude with one observation on the question of the localisation of function in the cerebellum. Is there any evidence that one part of the cerebellum is more connected with any one function, say that of walking, than is another? It has been suggested that by observations on the anatomy of the cerebellum in various kinds of animals, in some of whom the hind legs are better developed than the forelegs, or *vice versa*, it may be possible to assign to the divisions of the cerebellum control over definite regions of the body. In my opinion, such an application of anatomy to the deduction of physiological function is quite unjustifiable. There is, however, one division of the cerebellum, the vermis, which is certainly connected anatomically and structurally with the spinal cord. Disturbances after lesions of different parts of the vermis are due to disturbances of the intrinsic nuclei of the cerebellum. But to deduce the minute localisation of function in this part of the brain is at present, I think, a mistake.

